# Validation of the Mother-to-Infant Bonding Scale for Infant Maltreatment by Mothers at One Month Postpartum: An Adjunct Study of the Japan Environment and Children’s Study

**DOI:** 10.31662/jmaj.2022-0041

**Published:** 2022-06-17

**Authors:** Toshie Nishigori, Taku Obara, Hirohito Metoki, Kasumi Sakurai, Miyuki Mori, Taeko Suzuki, Mami Ishikuro, Nozomi Tatsuta, Masatoshi Saito, Junichi Sugawara, Takahiro Arima, Kunihiko Nakai, Shinichi Kuriyama, Nobuo Yaegashi, Hidekazu Nishigori

**Affiliations:** 1 Department of Pharmaceutical Sciences, Tohoku University Hospital, Miyagi, Japan; 2 Environment and Genome Research Center, Tohoku University Graduate School of Medicine, Miyagi, Japan; 3 Tohoku Medical Megabank Organization, Tohoku University, Miyagi, Japan; 4 Division of Public Health, Hygiene and Epidemiology, Faculty of Medicine, Tohoku Medical and Pharmaceutical University, Miyagi, Japan; 5 Department of Development and Environmental Medicine, Fukushima Medical Center for Children and Women, Fukushima Medical University Graduate School of Medicine, Fukushima, Japan; 6 Department of Obstetrics and Gynecology, Tohoku University Graduate School of Medicine, Miyagi, Japan; 7 Department of Sport and Health Science, Tokai Gakuen University, Aichi, Japan; 8 International Research Institute for Disaster Science, Tohoku University, Miyagi, Japan

**Keywords:** Mother-to-Infant Bonding Scale, anger, rejection, infant maltreatment, adjunct study of the Japan Environment and Children’s Study

## Introduction

Bonding failure is a mental disorder among parents characterized by reduced affection and anger toward, rejection of, and impulse to harm the infant ^(1), (2)^. The Japanese version of the Mother-to-Infant Bonding Scale (MIBS-J) has been widely used as a screening tool for assessing mother-to-infant bonding failure at 1 month postpartum to ensure mother-infant care support so as to prevent infant maltreatment in Japan ^(3), (4), (5), (6)^. An MIBS-J overall score of 4/5 at 1 month postpartum was suggested as the cutoff score for indicating infant maltreatment by mothers ^(5)^. The MIBS-J has two subscales, namely, lack of affection and anger/rejection ^(3), (7)^. In the present study, we evaluated the validity of the overall MIBS-J and its subscales for infant maltreatment by mothers at 1 month postpartum as an adjunct to the Japan Environment and Children’s Study (JECS) ^(8), (9)^.

## Methods

### Design and participants

The JECS is a nationwide prospective birth cohort study. It includes 100,000 pairs of mothers and offspring ^(8), (9)^. The JECS protocol has been previously published ^(8), (9)^. Participants who lived in the Miyagi Prefecture were further recruited to participate in this adjunct study ^(4)^. The recruitment period for the JECS was from January 2011 to March 2014, whereas that for the adjunct study was from August 2012 to November 2014 ^(4)^.

This study was conducted in accordance with the principles of the Declaration of Helsinki. It was also reviewed and approved by the Ministry of the Environment’s Institutional Review Board on Epidemiological Studies (Reference no. 100910001) and the Ethics Committee of Tohoku University Graduate School of Medicine (Reference no.: 2021-1-187). Written informed consent was obtained from all participants.

### Mother-to-infant bonding

The MIBS-J questionnaire is a self-report scale containing ten items scored on a four-point Likert scale. The MIBS-J has two subscales, namely, lack of affection (MIBS-J_LA: items 1, 6, 8, and 10) and anger and rejection (MIBS-J_AR: items 2, 3, 5, and 7) ^(3), (7)^. High scores indicate high severity of bonding failure. The MIBS-J questionnaire was mailed to mothers who agreed to participate in this adjunct study at 1 month postpartum ^(4)^.

### Infant maltreatment

In the JECS, the mothers were asked regarding the frequency to which they engaged in the following behaviors related to infant maltreatment at 1 month postpartum.

1. Frequency of leaving the baby alone at home

2. Frequency of ignoring the baby when he/she cries

3. Frequency of hitting the baby

Each statement consisted of four options: 1) often, 2) sometimes, 3) seldom, and 4) never. In this study, the responses “*seldom*,” “*sometimes*,” and “*often*” reflected infant maltreatment. It should be noted that there is no law in Japan that punishes caregivers for leaving infants alone at home.

### Statistical analysis

Receiver operating characteristic (ROC) curves were drawn based on the MIBS-J, MIBS-J_LA, and MIBS-J_AR scores. The area under the ROC curves (AUC) was measured to evaluate the scores obtained on the MIBS-J, MIBS-J_LA, and MIBS-J_AR. The optimal cutoff point was verified for items with an AUC greater than 0.7 and the largest AUC in infant maltreatment by mothers. To determine the optimal cutoff point, we used Youden’s index―which maximizes the sum of sensitivity and specificity―and considered the minimum distance from the upper-left corner on the ROC curve, which was calculated as {(1-sensitivity)^2^ + (1-specificity)^2^ }^1/2^.

Statistical analyses were conducted using the IBM SPSS Statistics 27 software (IBM Corp., Armonk, NY, USA).

## Results

A total of 9,217 pregnant women (mothers) in the Miyagi Prefecture participated in the JECS study between January 2011 and March 2014. The number of mothers requested to participate in this adjunct study was unclear; thus, the rate of recruitment could not be calculated.

Questionnaires were sent to 3,556 women at 1 month postpartum between August 2012 and November 2014; 3,294 responses (92.6%) were received. For this study, we analyzed data from mothers who had undergone only one delivery. In total, 3,225 participants responded to all items in the MIBS-J questionnaire and were thus included in this study. The participants (M_age_ = 30.5 years, SD = 5.1 years) consisted of 1,185 primiparous and 2,002 multiparous mothers (38 mothers had no answer for parity).

**Figure 1.**
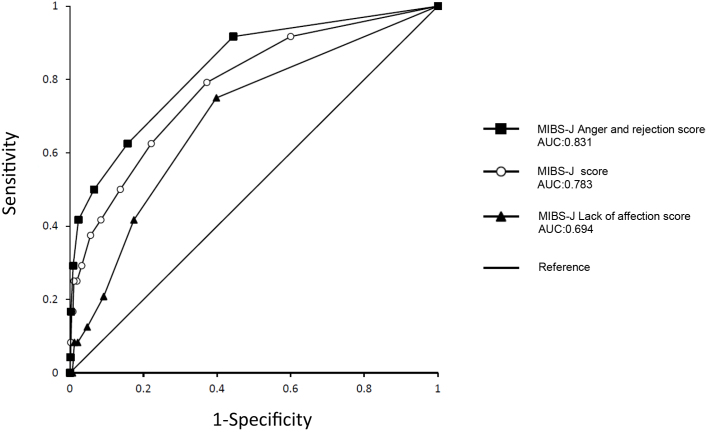
ROC curve for “hitting the baby” based on the MIBS-J scores at 1 month postpartum.

Of the 3,225 mothers, 440 (13.6%) left their baby alone at home, 2,096 (65%) ignored the baby when he/she cried, and 24 (0.7%) hit the baby. The ROC curve was generated for each item, and the AUC of each item is presented in Table 1. The ROC curve for *hitting the baby* based on the MIBS-J score is presented in Figure 1. The sensitivity and specificity for “hitting the baby” based on the MIBS-J_AR score (AUC = 0.831) are presented in Table 1. The optimal MIBS-J_AR cutoff score for “hitting the baby” based on Youden’s index (0.473) was 0/1, whereas that based on the shortest distance from the upper-left corner (0.407) was 1/2 (Table 2).

**Table 1.** AUC for Infant Maltreatment by Mothers.

Infant maltreatment by mothers	MIBS-J score	MIBS-J_LA score	MIBS-J_AR score
	AUC	AUC	AUC
Leaving the baby alone at home	0.526	0.521	0.519
Ignoring the baby when he/she cries	0.602	0.590	0.562
Hitting the baby	0.783	0.694	0.831

AUC, Area under the receiver operating characteristic curves MIBS-J, Japanese version of Mother-to-Infant Bonding Scale LA, Lack of affection AR, Anger and rejection

**Table 2.** Sensitivity, Specificity, Youden’s Index, and Distance from the Upper-left Corner for each Cutoff Score on the MIBS-J Anger and Rejection Subscale with reference to “hitting the baby”.

Cutoff score	n	%	Sensitivity	Specificity	Youden’s Index	Distance to the upper-left corner
0	1782	55.3	1.000	0.000	0.000	1.000
1	926	28.7	0.917	0.556	0.473	0.452
2	294	9.1	0.625	0.843	0.468	0.407
3	138	4.3	0.500	0.934	0.434	0.504
4	49	1.5	0.417	0.977	0.394	0.583
5	23	0.7	0.292	0.991	0.283	0.708
6	6	0.2	0.167	0.997	0.164	0.833
7	3	0.1	0.042	0.998	0.040	0.958
8	1	0	0.042	0.999	0.041	0.958
9	1	0	0.042	0.999	0.041	0.958
12	2	0.1	0.000	0.999	−0.001	1.000

MIBS-J, Japanese version of Mother-to-Infant Bonding Scale Youden’s Index, sensitivity + specificity −1 Distance to the upper-left corner; {(1-sensitivity)^2＋^(1-specificity)^2^}^1/2^

## Discussion

In this study, the MIBS-J_AR score for *hitting the baby* had the largest AUC, which was greater than 0.8. A cutoff score of 0/1 (based on Youden’s index) or 1/2 (based on the shortest distance from the upper-left corner) was considered to be optimal for the MIBS-J_AR score (Table 1). As the cutoff scores of 0/1 and 1/2 had similar Youden’s indices (0.473 and. 0.468, respectively), the cutoff score of 1/2 might be optimal at 1 month postpartum. The AUC of the other two items (*leaving the baby alone at home* and *ignoring the baby when he/she cries*) based on the overall MIBS-J score, MIBS-J_LA score, and MIBS-J_AR score did not exceed 0.7.

The overall score of the MIBS has been traditionally used as a clinical indicator ^(3), (6), (10)^. An MIBS-J overall score of 4/5 at 1 month postpartum was suggested as the cutoff score for indicating infant maltreatment ^(5)^. However, the MIBS-J has a two-factor structure of lack of affection and anger/rejection; therefore, the overall score should be evaluated in accordance with the respective subscales ^(3), (7)^. In fact, our results indicated that the overall score of 4/5 ^(5)^ is as not suitable as the MIBS-J_AR cutoff score of 1/2 for indicating the risk of physical infant maltreatment in terms of specificity and sensitivity.

This study has certain limitations. The participants of the JCES volunteered to participate in this adjunct study, which led to a selection bias. As it was a questionnaire-based study, the participants may not have honestly responded to the MIBS-J and infant maltreatment. Thus, the results might not reflect actual infant maltreatment by mothers. However, to the best of our knowledge, this is the first study to examine the validity of the overall MIBS-J and its subscales for infant maltreatment by mothers.

## Conclusions

The MIBS-J_AR is appropriate for assessing the risk of physical maltreatment of infants by mothers at 1 month postpartum on account of mother-to-infant bonding failure. For “hitting the baby,” the optimal cutoff score was 1/2 based on minimum distance to the upper-left corner. The results indicated that it is necessary to provide support for mothers based on subscale scores of the MIBS-J even when the overall MIBS-J score is not high; this may help combat and/or prevent the worsening of infant maltreatment.

## Article Information

### Conflicts of Interest

None

### Sources of Funding

The Japan Environment and Children’s Study was funded by the Ministry of the Environment, the Government offices of Japan. The findings and conclusions of this article are solely the responsibility of the authors and do not represent the official views of the aforementioned government. This adjunct study was supported by JSPS KAKENHI (C) Grant no. 24592457, the Mental Health Okamoto Memorial Foundation, and Fukushima Research Fund for Doctors in Specific Departments Grant no. R1-1.

### Acknowledgement

The authors are grateful to all the study participants; the research coordinators, Ms Miwa Kimura, Ms Atsuko Keino, Ms Rieko Tomizawa, and Mr Jun Memesawa; and the members of the JECS Miyagi Regional Centre for their expertise and guidance.

### Author Contributions

Authors’ contributions are as follow: T.N., T.O., H.M., and H.N. designed the study. T.N., T.O., H.M., K.S., M.I., T.A., K.N., S.K., N.Y., and H.N. conducted the study. T.N. M.M., T.S., and H.N. analyzed the data. T.N., T.O., H.M., K.S., M.M., T.S., M.I., T.N., M.S., J.S., T.A., K.N., S.K., N.Y., and H.N. interpreted the findings. T.N. and H.N. wrote the manuscript. All authors approved of the final draft.

### Approval by Institutional Review Board (IRB)

The Ministry of the Environment’s Institutional Review Board on Epidemiological Studies (Reference no. 100910001).

Ethics Committee of Tohoku University Graduate School of Medicine (Reference no.: 2021-1-187).

### Informed Consent

Written informed consent was obtained from all participants.
